# Electrochemical dissolution of steel as a typical catalyst for electro-Fenton oxidation

**DOI:** 10.1007/s00706-016-1688-8

**Published:** 2016-02-22

**Authors:** Veronika Kočanová, Libor Dušek

**Affiliations:** Institute of Environmental and Chemical Engineering, Faculty of Chemical Technology, University of Pardubice, Pardubice, Czech Republic

**Keywords:** Fenton reaction, Corrosion, Alloys, Metals, Electrochemistry

## Abstract

**Abstract:**

Although traditional Fenton reaction is known for a long time, it is still a perspective method for removal of pollution from wastewater. Applications of electro-Fenton oxidation are commonly used in wastewater treatment. These methods are classified into groups—electrochemical advanced oxidation processes. Typical catalysts for these technologies are Fe^2+^ ions. Comparison between two material types of steel was investigated in this paper. Alloy steel Cr–Ni and non-alloy steel were used as a source of Fe^2+^ ions as catalyst for electro-Fenton oxidation. Electrochemical dissolution was chosen as a method of catalyst dosage. Various parameters were tested depending on the type of material of alloy and non-alloy steel at a time. Corrosion properties were also experimentally tested of both materials of steel anodes. Electrochemically dissolved Fe^2+^ and Fe^3+^ sludge could be very well removed from treated water by the sedimentation process. At first the solutions were adjusted, then loosely precipitated, and at the end sedimented. Residual concentrations of iron in the solutions determined by UV/VIS spectrophotometry were in compliance with the threshold limits stated by the government regulation.

**Graphical abstract:**

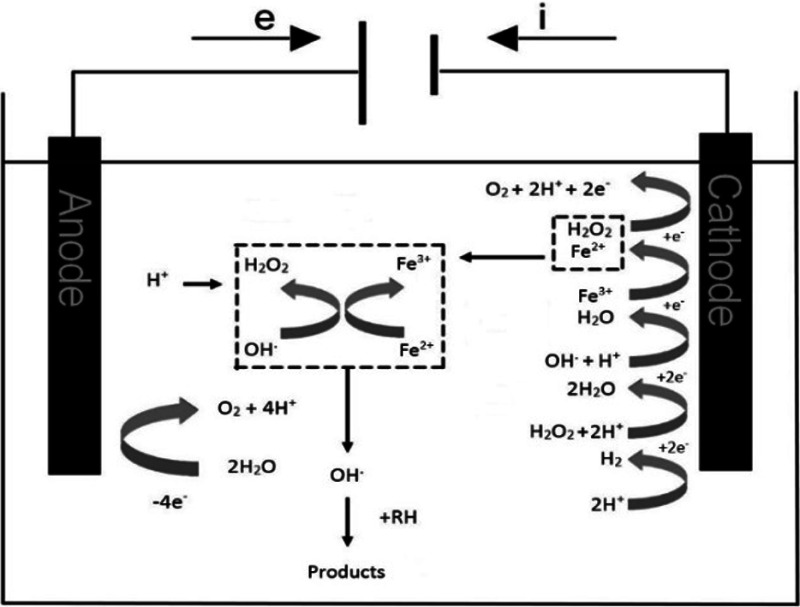

## Introduction

**Fig. 1:**
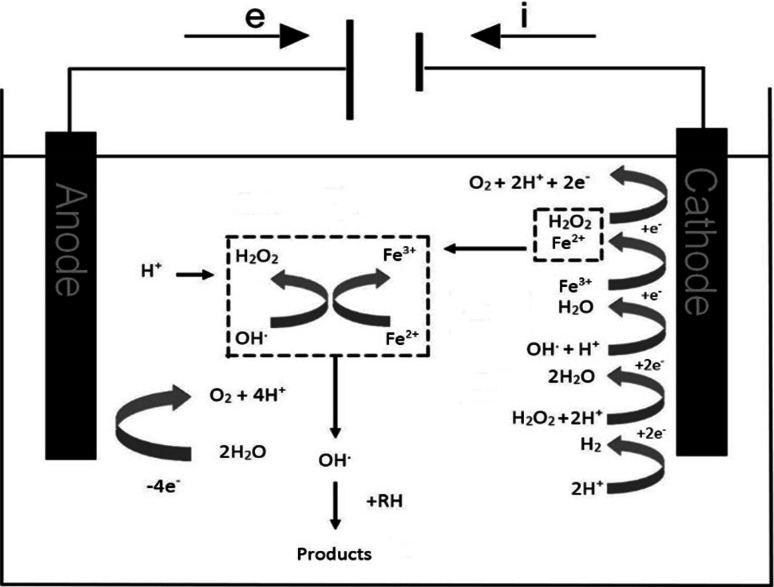
Oxidation of organic pollutants by the electro-Fenton process

In terms of wastewater treatment, commonly used techniques are based on Fenton, respectively electro-Fenton reaction. These techniques are included in electrochemical advanced oxidation processes (EAOPs) [1]. They could be applied to different types of contaminants of wastewater. The method of electro-Fenton oxidation is applied, especially in wastewater which is polluted by organic substances; see Fig. 1. It can remove drugs, phenol compounds, dyes, pesticides, and herbicides from the treated water [2]. It is also possible to purify water and soil from metals, such as for e.g., Pb, Cd, Cr, As, Mn, Cu, Zn, Ni, Al, Fe, Co, Sn, Mg, Se, Mo, Ca, and Pt with the electro-Fenton process [3, 4], as well as the anions CN^−^, PO_4_^3−^, SO_4_^2−^, NO^3−^, F^−^, or Cl^−^. From the group of non-metals, P could also be removed from wastewater [3]. Threshold limits for pollutants in drinking water and wastewater are enforced on the national and international scale [5, 6]. Electricity has been used for wastewater treatment from 1889, but electrochemical techniques are still considered to be simple and effective methods with compact size of the equipment and low capital and operating costs [7].

Fe^2+^ ions are a typical catalyst for electro-Fenton oxidation. However, in special applications, other types of catalysts, transition of metal ions, could be used [8]. Iron can be dosed in a form of a solution or in a solid phase. The most frequently used source of ferrous ions is FeSO_4_·7H_2_O. Oxides of iron or Fe^0^ applied on a carrier could be supplied in the solid phase. Fe^2+^ ions could be dosed using a sacrificial steel, iron or cast iron anode. Alternatively, these materials could be exposed to acidic non-oxidizing medium. The source of Fe^2+^ ions could be applied in an electrochemical concentration cell [9].

The efficiency of the electro-Fenton process depends on many factors, such as pH, temperature, concentration and form of catalyst (Fe^2+^), concentration of H_2_O_2_, current density, H_2_O_2_/Fe^2+^ and treatment time [1, 10]. The highest efficiency of Fenton oxidation is achieved in the pH range 2–4 [1].

Electro-Fenton oxidation is based on Fenton reaction, where in the first reaction Fe^2+^ reacts with hydrogen peroxide in acidic medium to give the hydroxyl radical and Fe^3+^; see Eq. (1).

1 $${\text{Fe}}^{ 2+ } + {\text{H}}_{ 2} {\text{O}}_{ 2} \to {\text{Fe}}^{ 3+ } + {\text{OH}}^{ - } + {\text{OH}}^{\cdot} .$$

During electro-Fenton oxidation, hydrogen peroxide is formed by the reduction of dissolved oxygen on the cathode. Afterward, the peroxide reacts with added ferrous ions to form a hydroxyl radical. The generated peroxide reacts with added ferrous ions to form a hydroxyl radical as in Eq. (2):

2 $${\text{O}}_{ 2} + 3 {\text{H}}^{ + } + 3 {\text{e}}^{ - } \to {\text{H}}_{ 2} {\text{O}} + {\text{OH}}^{\cdot} .$$

Ferrous ions are regenerated by reducing ferric ions on the surface of the cathode as in Eq. (3):

3 $${\text{Fe}}^{ 3+ } + {\text{e}}^{ - } \to {\text{Fe}}^{ 2+ } .$$

Oxidation of water occurs in the anodic area simultaneously with acidification around the anode and oxygen is released, as in Eq. (4). The formation of hydroxyl radical proceeds according to Eq. (5):

4 $${\text{H}}_{ 2} {\text{O}} \to \raise.5ex\hbox{$\scriptstyle 1$}\kern-.1em/ \kern-.15em\lower.25ex\hbox{$\scriptstyle 2$}\, {\text{O}}_{ 2} + 2 {\text{ H}}^{ + } + 2 {\text{ e}}^{ - } ,$$

5 $$\raise.5ex\hbox{$\scriptstyle 1$}\kern-.1em/ \kern-.15em\lower.25ex\hbox{$\scriptstyle 2$} \,{\text{O}}_{ 2} + {\text{H}}_{ 2} {\text{O}} \to 2 {\text{OH}}^{\cdot} .$$

Hydroxyl radicals are generated in the presence of water, oxygen, and power supply [11].

## Results and discussion

Two types of material—Steel 17 240 Cr–Ni (DIN X 5 CrNi 18 10, AISI 304) and Steel 11 373 (DIN USt 37-2)—were experimentally tested in the form of two types of steel anodes. The catalyst was dosed by electrochemical dissolution of these sacrificial anodes.

The amount of dissolved iron in oxidation state II as a catalyst was determined by absorption spectrophotometry. Fe^2+^ ions react with 1,10-phenanthroline to form a red-orange complex which is most stable at pH 3–4. Total dissolved Fe was determined gravimetrically. It was possible to obtain the current amount of the catalyst during 15 min after stabilization of the colored complex and uninstallation of the active part of a sacrificial steel anode. The benefit is that at the same time, the current concentrations of Fe^2+^ and total iron were observed. Experiments were run at constant pH 4 under a current of 0.010 A, 0.025 A, 0.050 A, 0.100 A, and 0.150 A. Experimental conditions were identical for both of those tested materials of the sacrificial anode.

**Fig. 2:**
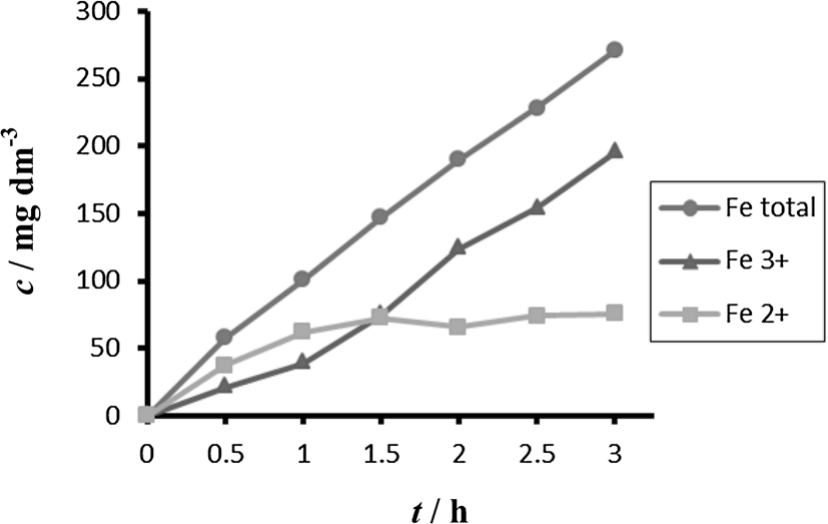
Time dependence of concentration of Fe total, Fe^2+^, and Fe^3+^ for anode (DIN USt 37-2), pH 4, *I* = 0.010 A, *t* = 25 °C

**Fig. 3:**
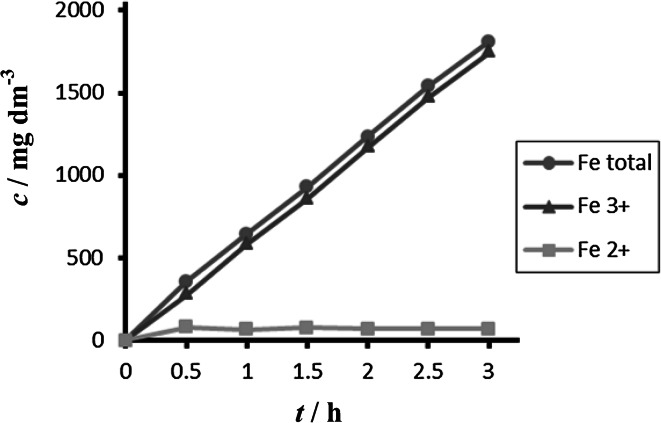
Time dependence of concentration of Fe total, Fe^2+^, Fe^3+^ for anode (DIN USt 37-2), pH 4, *I* = 0.150 A, *t* = 25 °C

The obtained concentrations of all forms of iron during electrochemical dissolution of non-alloy steel depending on time and different current densities are summarized in Figs. 2 and 3.

**Fig. 4:**
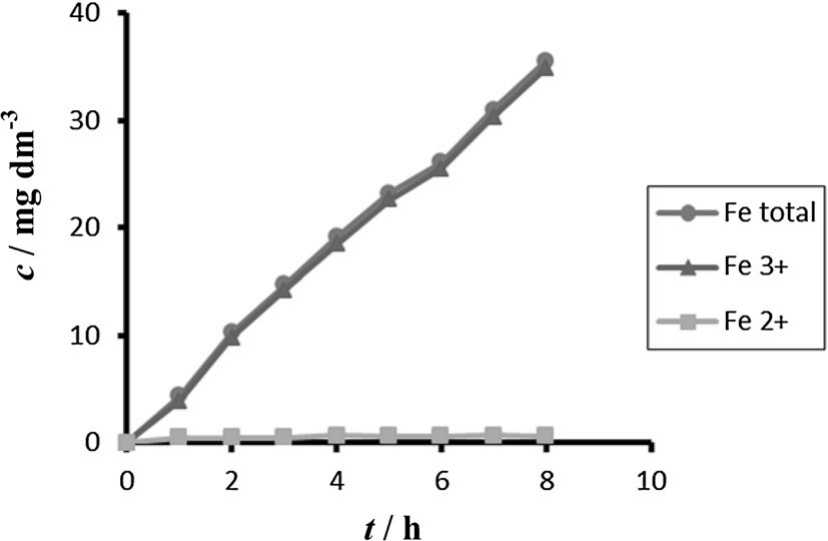
Time dependence of concentration of Fe total, Fe^2+^, Fe^3+^ for anode (DIN X 5 CrNi 18 10, AISI 304), pH 4, *I* = 0.025 A, *t* = 25 °C

**Fig. 5:**
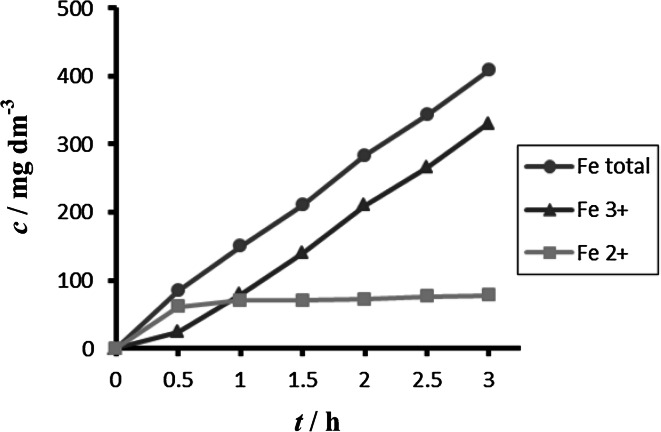
Time dependence of concentration of Fe total, Fe^2+^, Fe^3+^ for anode (DIN USt 37-2), pH 4, *I* = 0.025 A, *t* = 25 °C

**Fig. 6:**
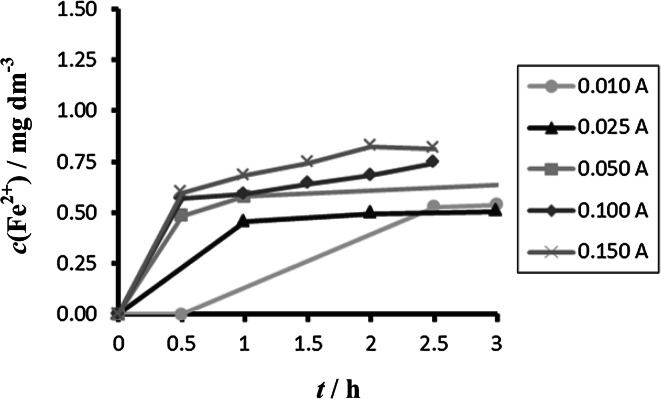
Time dependence of concentration of Fe^2+^ for anode (DIN X 5 CrNi 18 10, AISI 304), pH 4, *I* = 0.010–0.150 A, *t* = 25 °C

**Fig. 7:**
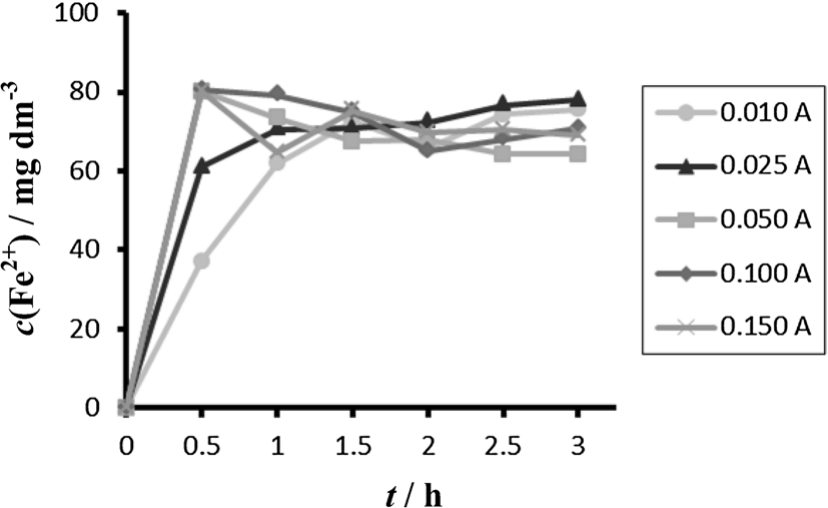
Time dependence of concentration of Fe^2+^ for anode (DIN USt 37-2), pH 4, *I* = 0.010–0.150 A, *t* = 25 °C

**Fig. 8:**
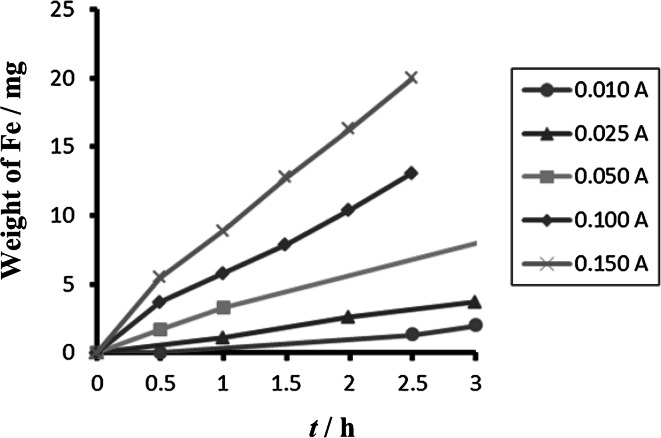
Time dependence of weight losses of anode (DIN X 5 CrNi 18 10, AISI 304), cathode Pt, pH 4, *I* = 0.010–0.150 A, *t* = 25 °C

**Fig. 9:**
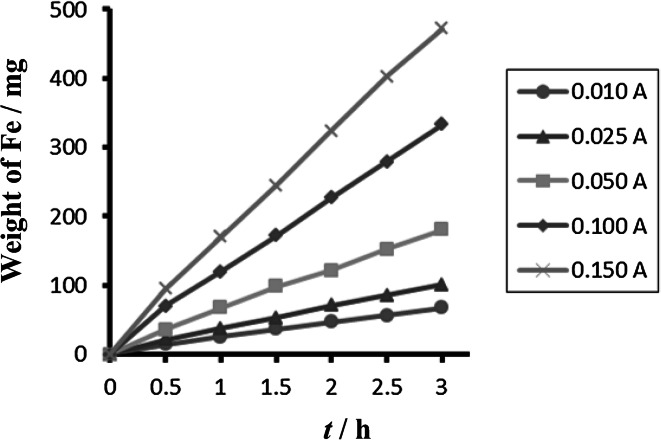
Time dependence of weight losses of anode (DIN USt 37-2), cathode Pt, pH 4, *I* = 0.010–0.150 A, *t* = 25 °C

Comparison between different tested materials depending on time is shown in Figs. 4 and 5. Concentrations of Fe^2+^ were very low in comparison to total iron. Dependence of concentration of Fe^2+^ on time is shown in Figs. 6 and 7. The total weight losses of iron anode were determined by the gravimetric method. Figures 8 and 9 show weight losses of the iron sacrificial anode during the duration of the experiment.

Corrosion tests without electric current were conducted. This experiment demonstrated that the Steel 17 240 Cr–Ni (DIN X 5 CrNi 18 10, AISI 304) without current nearly does not dissolve. It can be concluded that the influence of atmospheric oxygen is negligible. The corrosion losses of alloy steel Cr–Ni are fourteen hundred times lower than non-alloy steel at pH 4. The corrosion rates of alloy steel Cr–Ni are twelve thousand times lower than non-alloy steel at pH 4; see Tables 1 and 2. The corrosion rates could be determined from the corrosion losses. Commonly, we relate corrosion rates to years [12].

**Table 1:** Corrosion test without electric current—alloy steel Cr–Ni

pH	t/h	Total loss/mg	Corrosion loss/g m^−2^	Corrosion rate/g m^−2^ y^a^
2	505.5	0.3	0.26	4.48
3	505.5	0.2	0.17	2.99
4	505.5	0.1	0.09	1.49

^a^y = per year

**Table 2:** Corrosion test without electric current–non-alloy steel

pH	t/h	Total loss/mg	Corrosion loss/g m^−2^	Corrosion rate/g m^−2^ y^a^
2	61	492.7	425.02	61035.23
3	61	194.7	167.95	24119.26
4	61	148.2	127.84	18358.88

^a^y = per year

Ferrous and ferric sludge could be removed from solutions by sedimentation. At first, solutions were adjusted to pH 7.5 and 10. Then, ions were precipitated and then sedimented. The concentrations of total iron of 0.38 and 0.37 mg dm^−3^ in the collected samples were in compliance with the government regulation (No. 61/2003 Coll.) for values of pollution by iron in the surface and wastewater, where the limit for iron is 0.55 mg dm^−3^.

After finishing experiments with Steel 17 240 Cr–Ni, the quantitative analysis of Fe, Cr, and Ni was done with the inductively coupled plasma optical emission spectrometry (ICP-OES) technique. The results of the ICP-OES analysis of the total concentration of Fe, Ni, and Cr after 2.5 h of electrochemical dissolution of a sacrificial anode Steel 17 240 Cr–Ni with a weight loss Δ*m* in 250 cm^3^ single-chamber electrolytic cell at temperature 25 °C, current *I*, and pH 2–4, respectively, after increase to values 7.5 and 10, are in Table 3.

**Table 3:** Total concentration of Fe, Ni, and Cr (mg dm^−3^) from the ICP-OES

*I/*A	Δ*m*_anode_^a^/mg	pH 2			pH 7.5			pH 10		
		*c*_Fe_	*c*_Ni_	*c*_Cr_	*c*_Fe_	*c*_Ni_	*c*_Cr_	*c*_Fe_	*c*_Ni_	*c*_Cr_
0.010	7.66	21.89	2.87	5.46	0.15	2.44	0.23	0.11	2.09	>0.01
0.025	14.83	42.38	5.61	10.62	0.27	4.82	0.27	0.22	4.75	>0.01
0.050	33.70	96.31	12.77	24.34	0.34	8.78	0.43	0.14	5.47	0.05
0.100	57.40	164.05	21.87	41.42	0.56	9.32	0.39	0.36	5.63	>0.01
0.150	71.91	205.52	27.31	51.66	0.38	10.62	0.48	0.37	5.94	0.03

*I/*A	Δ*m*_anode_^a^/mg	pH 3			pH 7.5			pH 10		
		*c*_Fe_	*c*_Ni_	*c*_Cr_	*c*_Fe_	*c*_Ni_	*c*_Cr_	*c*_Fe_	*c*_Ni_	*c*_Cr_
0.010	3.63	10.37	1.43	2.57	0.23	1.28	0.19	0.14	1.15	>0.01
0.025	7.83	22.38	2.95	5.61	0.27	2.30	0.25	0.19	2.02	>0.01
0.050	23.08	65.96	8.72	16.64	0.26	7.12	0.23	0.12	4.67	0.02
0.100	26.80	76.59	10.23	19.36	0.44	9.38	0.31	0.23	5.14	>0.01
0.150	59.70	170.62	22.72	43.05	0.30	10.21	0.44	0.18	6.06	0.02

*I/*A	Δ*m*_anode_^a^/mg	pH 4			pH 7.5			pH 10		
		*c*_Fe_	*c*_Ni_	*c*_Cr_	*c*_Fe_	*c*_Ni_	*c*_Cr_	*c*_Fe_	*c*_Ni_	*c*_Cr_
0.010	1.30	3.72	0.43	0.92	0.17	0.19	0.14	0.07	0.13	0.01
0.025	3.25	9.29	1.21	2.36	0.31	1.19	0.09	0.16	0.97	>0.01
0.050	8.25	23.58	3.17	5.99	0.41	2.73	0.11	0.26	2.47	>0.01
0.100	13.10	37.40	5.03	9.51	0.35	4.20	0.18	0.32	3.22	0.04
0.150	20.01	57.16	7.57	14.48	0.36	7.31	0.26	0.23	6.12	0.07

^a^Δ*m*_anode_, weight loss of sacrificial anode

## Conclusion

Electrochemical advanced oxidation processes (EAOPs) which are used in wastewater treatment could be based on electro-Fenton oxidation. Fenton reaction has been well known and commonly used for several years, but it is still an effective method for removal of different pollutants from wastewater. In this paper, two types of materials of typical catalyst for Fenton reaction were tested. Catalyst in form of ions of Fe^2+^ is used because it achieves the highest efficiency. It is relatively inexpensive, non-toxic and well regenerable and removable from treated water. Electrochemical dissolution of sacrificial steel anode was tested as an effective method of catalyst dosage. During laboratory experiments, two different types of material steel anode were compared. Alloy steel–steel 17 240 Cr–Ni (DIN X 5 CrNi 18 10, AISI 304) and non-alloy steel–steel 11 373 (DIN USt 37-2) were chosen. Both materials are commonly commercially available products with excellent constant quality. But alloy steel Cr–Ni is five times more expensive than non-alloy steel. The influence of pH in the range of 2–4 was tested. For application of alloy steel, Cr–Ni connection to DC power supply is necessary to increase the low corrosion rate. Non-alloy steel is susceptible to a rapid corrosion process. The corrosion rates obtained after recalculation were 18.36 kg m^−2^ year (pH 4); 24.12 kg m^−2^ year (pH 3) to 61.04 kg m^−2^ year (pH 2). Alloy steel Cr–Ni shows corrosion losses of about 1 420–1 640 times lower, which corresponds with the corrosion rate of 1.5 × 10 ^−3^ kg m^−2^ year at pH 4. Steel Cr–Ni can be considered as a well-regulated source of catalyst for electro-Fenton applications. Conversely, non-alloy steel shows enough corrosion rate to dose ions Fe^2+^ to the electro-Fenton process, but it is characterized by low level of regulation of the whole process without electric current. Consequently, the concentration of ferrous and ferric ions increases. Sedimentation could be an effective method for the removal of ferrous and ferric sludge. Only residual concentrations of iron were detected and the collected samples were in compliance with the conditions stated in the government regulation. The efficiency of electro-Fenton oxidation depends on a suitable pH (2–4) and an optimal ratio of H_2_O_2_:Fe^2+^. The results of our experiments show that a precise dosage of Fe^2+^ with sacrificial steel anode of Steel 11 373 (DIN USt 37-2) in the concentration range of 0.2–5.0 mg dm^−3^ is not achievable. The corrosion rate of Steel 11 373 (DIN USt 37-2) is too high in the above-mentioned pH range even without electric current. Therefore, this material needs to be excluded as a source of Fe^2+^ for catalysis of electro-Fenton reaction in an environment with low concentration of organic pollutants. On the contrary, fast availability of high concentration of an active form of iron allows intensification of the electro-Fenton process during industrial applications with high concentrations of organic, biologically resistant pollutants. The noticeable advantage of Steel 11 373 (DIN USt 37-2) is its low price, less than 1 €/kg and good availability (even iron crap can be used). In those cases, iron in all of its forms can be removed simply by neutralization of treated water and followed by sedimentation of the resulting sludge. The determined residual concentration of total iron of 0.38 mg dm^−3^ allows to continue using this water in industry or release it to surface water; see Table 4 [5, 13]. Sacrificial steel anode Steel 17 240 Cr–Ni (DIN X 5 CrNi 18 10, AISI 304) allows the precise dosage of Fe^2+^ ions in environmental applications of electro-Fenton oxidation. An example of its application could be its use in dismantling biologically resistant pharmaceuticals and personal care products in low concentrations at small residential sewage treatment plants [9]. The ions Cr^3+^ and Ni^2+^ were released into solution together with catalyzing Fe^2+^ ions during electrochemical dissolution. The molar ratio Fe^2+^:Cr^3+^:Ni^2+^ with pH range 2–4 corresponds to the presence of these elements in Steel 17 240 and their concentration to corrosion loss is given by the volume of treated water. Even after neutralization, i.e., rise of pH to 10, the creation of precipitate was observed. After removal of the precipitate (pH 7.5), the concentration of iron was 0.37 mg dm^−3^. The maximal concentration of nickel, experimentally provided with ICP-OES, was 10.62 mg dm^−3^, which corresponds with the formation of NiCO_3_ [14]. Its solubility in water is limited by a value of p*K*_s_^25^ (solubility at 25 °C) 6.87. The maximal concentration of chrome was under those conditions lower than 0.01 mg dm^−3^. This suggests the formation of Cr(OH)_3_, whose solubility in water is limited to p*K*_s_^25^ 30.20. After increase of the pH to 10, the maximal concentration of nickel decreased to 5.87 mg dm^−3^. The concentration of iron and chrome stayed unchanged. This phenomenon can be explained as an enervation of CO_2_ presented in water and by creation of Ni(OH)_2_ in alkaline range of pH, with its p*K*_s_^25^ 13.79. As mentioned above, it is obvious that neutralization of treated water is suitable for removing iron and chrome, not because of nickel, whose concentration enormously exceeded limits, under pH 10. A high concentration of nickel has no influence on the environmental application mentioned in the patent [9]. In this case, nickel is preferably deducted by the present phosphates, which in the case of small residential sewage treatment plants removed only by 40–80 %. The usual concentration of total phosphorus at the outlet of those plants is circa 6–12 mg dm^−3^. This is enough for residual precipitation of nickel, because p*K*_S_^25^ of nascent Ni_3_(PO_4_)_2_ is 30.30. This responds to the residual concentration of nickel of 3.2 µg dm^−3^, which is below the limits for drinking water (see Table 4) [5, 13]. In other applications, sorbents or ion exchangers are used for solvent problems [15–18].

**Table 4:** Threshold limits of metals in different types of waters in the Czech Republic

Metal	Concentration limits of metals/mg dm^−3^			
	Decree No. 252/2004 Coll. for drinking water	Gov. Reg. No. 61/2003 Coll. for surface water		Gov. Reg. No. 61/2003 Coll. for industrial wastewater
Cr	0.05	0.015	0.018	0.1–0.5
Ni	0.02	–	0.020	0.5–0.8
Fe	0.20	0.55	1	2–8

*Gov. Reg.* government regulation

**Table 5:** Chemical composition of alloy steel Cr–Ni

Chemical composition (analysis of meltage)/%	C	Mn	Si	Cr	Ni	P	S
	Max. 0.07	Max. 2	Max. 1	17.0–20.0	9.0–11.5	Max. 0.045	Max. 0.030
Permissible variations of chemical composition in final product/%	+0.01	+0.15	+0.05	+0.5	+0.5		
				−0.3	−0.3

**Table 6:** Chemical composition of non-alloy steel

Chemical composition (analysis of meltage)/%	C	P	S	N
	Max.	Max.	Max.	Max.
	0.17	0.045	0.045	0.07
Permissible variations of chemical composition in the final product/%	0.04	0.01	0.01	0.02

## Experimental

As sacrificial steel anodes, Steel 17 240 Cr–Ni (DIN X 5 CrNi 18 10, AISI 304) and Steel 11 373 (DIN USt 37-2) were tested. The average active area of anodes was 14 cm^2^. The chemical compositions of the steels used for experiments are given in Tables 1 and 2. The type of stainless Cr–Ni steel, according to the ČSN 41 7240 (the Czech norm), is characterized as austenitic, weldable, unstabilized and stainless steel. It is resistant to intergranular corrosion of welded metal sheets up to a thickness of about 6 mm [19] (Table 5).

The non-alloy steel is named as Steel 11 373 according to ČSN 41 1373 (the Czech norm). This plain structural steel is suitable for welding of steel structures. It could be a part of the construction and equipment of lower thicknesses which are a result of welding and static and dynamic test [20] (Table 6).

The cathode was made of platinum with a thickness of 0.3 mm and surface area of 1 cm^2^. The distance between the electrodes was 2 cm. The volume of the single-chamber electrolytic cell was 250 cm^3^. All experiments were carried out at constant laboratory temperature of 25 °C.

**Fig. 10:**
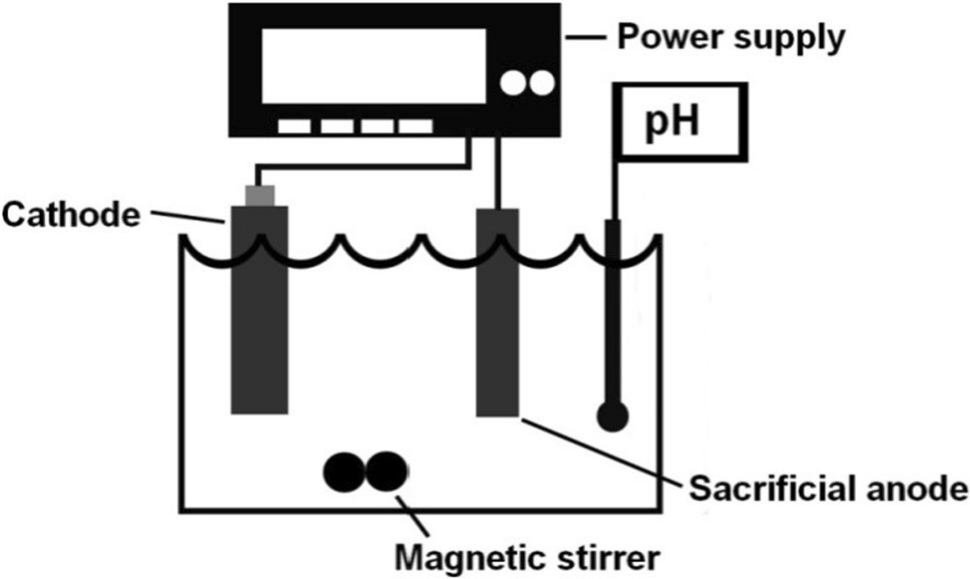
Experimental setup of the electrolytic cell

Experiments were carried out at pH index 2, 3, and 4. A constant value of pH was maintained using automatic titrator TitraLab 856 (Radiometer analytical, Lyon, France). For each level of pH, the amount of current was 0.010 A, 0.025 A, 0.050 A, 0.100 A, and 0.150 A. These levels of current were set on DC Power Supply SDP–2210 (Manson, Kwai Chung, N.T., Hong Kong). Experimental setup of the electrolytic cell can be seen in Fig. 10. Concentrations of Fe^2+^ were found out by the values of absorbance, which have been measured every 15 min since the creation of the color complex. After the reaction a with 1,10-phenanthroline, a spectrophotometric method was used for the determination of Fe^2+^. For measurement UV/VIS Spectrophotometer Libra S22 (Biochrom, Cambridge, UK) was used. Total amount iron was determined gravimetrically-the steel anode was continually weighted, Digital Analytical Balance 870 (Kern, Balingen, Germany). After finishing the experiment with Steel 17 240 Cr–Ni, the concentrations of Fe, Cr, and Ni were determined by ICP-OES Spectrometer Integra XL2 (GBC Scientific Equipment Pty Ltd., Australia). Iron is characterized by spectral lines 259.940 nm, chrome by 267.716 nm and nickel by 231.604 nm.
